# Repurposing of c-MET Inhibitor Tivantinib Inhibits Pediatric Neuroblastoma Cellular Growth

**DOI:** 10.3390/ph17101350

**Published:** 2024-10-09

**Authors:** Rameswari Chilamakuri, Saurabh Agarwal

**Affiliations:** Department of Pharmaceutical Sciences, College of Pharmacy and Health Sciences, St. John’s University, New York, NY 11439, USA

**Keywords:** tivantinib, ARQ197, c-MET, neuroblastoma, drug repurposing

## Abstract

**Background:** Dysregulation of receptor tyrosine kinase c-MET is known to promote tumor development by stimulating oncogenic signaling pathways in different cancers, including pediatric neuroblastoma (NB). NB is an extracranial solid pediatric cancer that accounts for almost 15% of all pediatric cancer-related deaths, with less than a 50% long-term survival rate. **Results:** In this study, we analyzed a large cohort of primary NB patient data and revealed that high *MET* expression strongly correlates with poor overall survival, disease progression, relapse, and high *MYCN* levels in NB patients. To determine the effects of c-MET in NB, we repurposed a small molecule inhibitor, tivantinib, and found that c-MET inhibition significantly inhibits NB cellular growth. Tivantinib significantly blocks NB cell proliferation and 3D spheroid tumor formation and growth in different MYCN-amplified and MYCN-non-amplified NB cell lines. Furthermore, tivantinib blocks the cell cycle at the G2/M phase transition and induces apoptosis in different NB cell lines. As expected, c-MET inhibition by tivantinib inhibits the expression of multiple genes in PI3K, STAT, and Ras cell signaling pathways. **Conclusions:** Overall, our data indicate that c-MET directly regulates NB growth and 3D spheroid growth, and c-MET inhibition by tivantinib may be an effective therapeutic approach for high-risk NB. Further developing c-MET targeted therapeutic approaches and combining them with current therapies may pave the way for effectively translating novel therapies for NB and other c-MET-driven cancers.

## 1. Introduction

Cancer therapy has significantly advanced in recent decades, particularly in the treatment of solid tumors, yet substantial challenges remain. Conventional approaches, including surgery, chemotherapy, and radiation, have improved survival rates for many patients but often result in severe side effects and the development of resistance, especially in aggressive tumors like neuroblastoma (NB). Emerging precise therapeutic approaches, including immunotherapy and gene editing, specifically target cancer cells and minimize side effects [1]. However, these therapies face notable limitations, such as immune evasion by tumors, genetic heterogeneity within solid tumors, and the high cost of personalized treatment strategies [1,2]. Addressing these challenges requires further research to enhance therapeutic efficacy, reduce toxicity, and improve access to advanced treatments.

NB is a common neuroendocrine solid tumor with highly diverse genetic, epigenetic, morphological, and biological characteristics, which complicates its diagnosis and treatment [3]. NB develops from neural crest cells of the peripheral nervous system and accounts for 6–10% of all pediatric cancers, with a five-year survival probability of less than 50% [4]. NB is characterized by significant genetic alterations, notably MYCN amplification and ALK mutations, which play pivotal roles in tumor initiation [3]. Similar to other cancer types, NB is driven by various regulatory and signaling pathways that contribute to neoplastic progression. NB exhibits marked heterogeneity, ranging from well-differentiated tumors, which are less aggressive, to undifferentiated forms, which are more malignant and associated with a poorer prognosis [1,3]. Targeted therapeutic approaches, including ALK inhibitors and anti-GD2 immunotherapy, have shown promising efficacy in improving clinical outcomes by addressing specific genetic mutations driving tumor growth [5]. Despite intensive multimodal therapeutic approaches, NB remains a clinical challenge with poor overall survival due to relapse, metastasis, and drug resistance [5]. Therefore, identifying and characterizing the novel molecular targets involved in NB oncogenesis and developing effective therapeutic targeted approaches are crucial for managing NB effectively.

Overexpression of different receptor tyrosine kinases has been reported to influence NB tumorigenesis, proliferation, angiogenesis, differentiation, and overall patient outcomes [6]. c-Met is a mesenchymal-epithelial transition factor and a proto-oncogene belonging to a family of MET receptor tyrosine kinases (RTKs). The liver, kidney, prostate, bone marrow, and pancreas epithelial cells express c-MET [7,8]. Ligands such as HGF (hepatocyte growth factor) directly bind and activate the c-MET receptor, and c-MET-HGF binding is responsible for wound healing, cell motility, embryogenesis, and muscle tissue development [8]. HGF binding leads to dimerization, autophosphorylation, and activation of the c-MET receptor, which further leads to the activation of a plethora of signaling pathways such as the PI3K/AKT, Ras/Raf, JAK/STAT, NF-κB, and GSK3α/β pathways [9,10]. Crosstalk between MET and other cell signaling pathways has appeared to be an essential mechanism for cancer development, metastasis, and drug resistance [11].

Dysregulation of the c-MET/MET pathway occurs through various mechanisms, including gene amplification, mutation, rearrangement, and protein overexpression, and has been reported to be involved in different cancers, including NB, NSCLC (non-small cell lung cancer), colorectal, ovarian, breast, gastric, and renal cancers [6,12]. In cases of non-hereditary cancer, somatic mutations in the MET gene are infrequent. Variations such as missense mutations and single nucleotide polymorphisms (SNPs) have been identified in two of the MET domains, including the SEMA and juxtamembrane domains [13]. Oncogenic point mutations lead to an altered splicing process that produces a truncated protein missing exon 14, responsible for the juxtamembrane domain of c-MET [14]. Mutations at Y1003 deactivate the Cbl binding site, causing over and constitutive c-MET expression [15]. Due to copy number variation, MET amplification results in higher MET protein levels and activates the downstream signaling pathways, including MAPK/ERK, PKC, and PI3K/AKT [15,16]. Irregular MET activity has been outlined to induce chemoresistance in various cancers and in the maintenance of refractory cancer stem cells, and downregulation of the c-MET/HGF pathway has been reported to reduce cancer stemness and overall tumor growth [17]. MET and HGF expression have been reported in various NB cell lines, and HGF-mediated cell invasion and angiogenesis were observed in NB tumor models [18]. As c-MET plays a crucial role in controlling multiple oncogenic pathways, developing direct targeting strategies for c-MET is an attractive curative approach for NB.

In this study, we used tivantinib, a specific c-MET inhibitor, to evaluate the role of MET inhibition in NB growth. Tivantinib (also called ARQ197) is a highly specific, non-competitive ATP inhibitor of c-MET RTK, which was developed by Kyowa Hakko Bio, Daiichi Sankyo, and ArQule Corporation [8,19]. Tivantinib directly binds to the c-MET receptor and disrupts the ionic interaction at the Tyr1234 catalytic residue site to inhibit ligand binding, phosphorylation, and receptor activation [8,20]. Tivantinib has been reported as a safe and well-tolerated drug in Phase 1 clinical trials in pediatric solid tumor patients, and the pharmacodynamic analysis reported an infrequent expression of c-MET. The association between treatment outcomes and tumor c-MET levels was constrained by factors such as low treatment effectiveness, rare occurrence of c-MET overexpression, and a small number of patients involved [21]. Tivantinib is shown to exert antitumor effects by microtubule disruption in a c-MET-independent manner [22,23,24]. These findings have led to debates about tivantinib’s classification as a c-MET inhibitor or an antimitotic agent [25,26].

Our data demonstrate that tivantinib inhibits c-MET phosphorylation and activation in NB, thereby inhibiting multiple downstream signaling cascades such as PI3K/AKT, JAK/STAT, and Ras/Raf pathways. Additionally, tivantinib significantly inhibited NB cell proliferation and colony formation. Further, we observed tivantinib-mediated induction of apoptosis and blockage of cell cycle progression in different NB cell lines. Tivantinib also significantly inhibited NB 3D spheroid tumor growth in multiple NB cell lines. Overall, our data indicate that c-MET signaling drives NB proliferation and 3D spheroid growth, and direct targeting of c-MET by tivantinib shows promising effects on NB cells. Further developing strategies to incorporate tivantinib along with ongoing therapies may provide an effective way of managing NB and other c-MET-driven cancers.

## 2. Results

### 2.1. MET Promotes NB Progression

**Figure 1:**
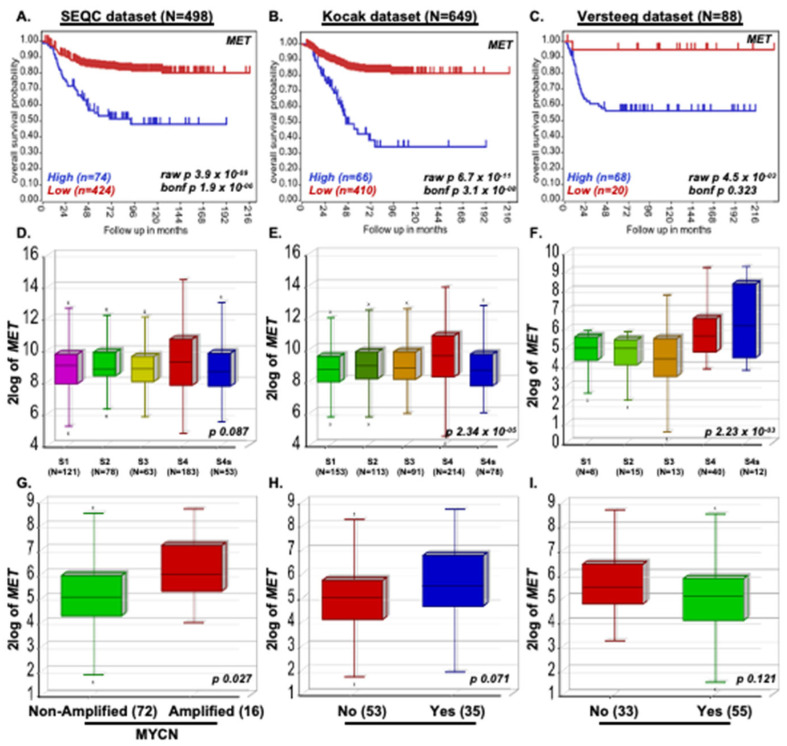
*MET* promotes NB Progression. Kaplan-Meier survival study shows that the MET expression correlates with poor overall survival of NB patients. (**A**) SEQC (n = 498), (**B**) Kocak (n = 649), (**C**) Versteeg (n = 88) dataset. (**D**–**F**) NB stage analysis demonstrating MET expression correlates with NB stage progression. (**D**) SEQC, (**E**) Kocak, (**F**) Versteeg dataset. (**G**–**I**) The Versteeg dataset showed that high MET expression correlates with (**G**) highly inimical MYCN-amplified tumors, (**H**) NB disease relapse conditions, and (**I**) worst outcomes.

We investigated the role of the MET gene in NB disease progression by analyzing 1235 NB primary patient data from three different patient datasets, including 88 patients in the Versteeg dataset, 649 patients in the Kocak dataset, and 498 patients in the SEQC dataset. Kaplan–Meier survival analysis demonstrated that elevated MET expression strongly correlates with poor overall survival of NB patients (Kocak p = 3.1 × 10^−8^; SEQC p = 1.9 × 10^−6^; Versteeg p = 0.323; Figure 1A–C). In all three datasets, we observed that higher-stage NB patients express higher levels of MET, suggesting that MET plays a significant role in NB stage progression (Kocak p = 0.087; SEQC p = 2.34 × 10^−5^; Versteeg p = 2.23 × 10^−3^; Figure 1D–F). We further observed that MET expression corresponds with high MYCN levels in MYCN amplified tumors (*p* = 0.027), disease relapse (*p* = 0.071), and worst outcome (*p* = 0.121) of NB patients in the Versteeg dataset (Figure 1G–I). These observations clearly highlight the role of MET in NB pathogenesis and progression. Our patient dataset analysis also suggests that developing direct targeting strategies for c-MET is a promising therapeutic approach for NB.

### 2.2. Tivantinib Inhibits NB Cell Proliferation

**Figure 2:**
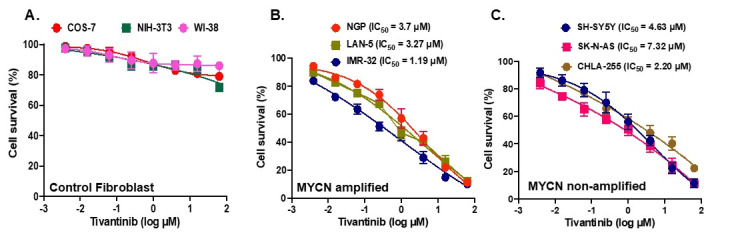
Tivantinib inhibits NB cell proliferation. Cytotoxic assays in response to tivantinib treatment. (**A**) Control fibroblast cell lines (WI-38, NIH-3T3, COS-7). (**B**) MYCN amplified cell lines (NGP, LAN-5, IMR-32). (**C**) MYCN non-amplified cell lines (SH-SY-5Y, SK-N-AS, CHLA-255). IC_50_ was calculated using a nonlinear variable slope regression method.

To evaluate the effects of MET inhibition in NB, we used tivantinib and performed cytotoxicity studies in various human NB and non-cancerous cell lines. We used three MYCN non-amplified NB cell lines, CHLA-255, SK-N-AS, and SH-SY5Y; three MYCN amplified NB cell lines, NGP, LAN-5, and IMR-32; three non-cancerous fibroblast cell lines, COS-7, NIH-3T3, and WI-38, for cytotoxicity studies (Figure 2). An analysis of the Cancer Cell Line Encyclopedia database revealed that NB cell lines, including NGP and SH-SY5Y, used in the present study for most of the experiments overexpress *MET*, and SH-SY5Y contains more copy numbers of MET in comparison to other NB cell lines (Data not included). Our cytotoxicity experiment data show that tivantinib significantly and in a dose-dependent manner inhibits cell proliferation in all six NB cell lines irrespective of their MYCN status while having a minimum effect on any fibroblast cell lines used (Figure 2). We observed a range of IC_50_ values among NB cell lines ranging from 1.19 µM for IMR-32 to 7.32 µM for SK-N-AS (Figure 2B,C). These data highlight the specific anti-proliferative effect of tivantinib for NB cancer cells in contrast to the non-cancerous human cells.

**Figure 3:**
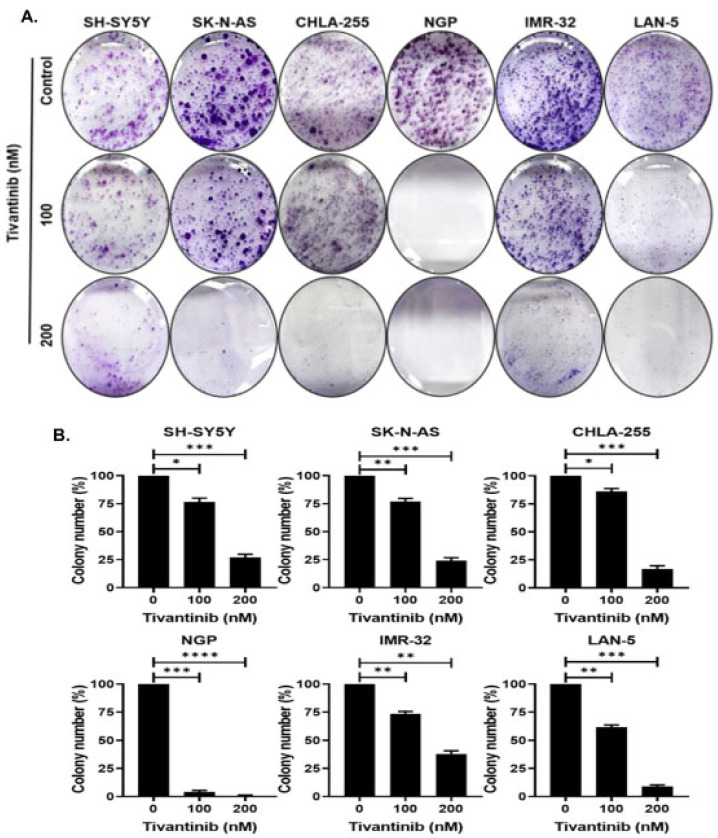
Tivantinib inhibits NB colony formation. Colony formation studies were conducted in response to tivantinib (**A**) Colony formation assay representative images in response to tivantinib in different NB cell lines. (**B**) Survival index graphs of individual NB cell lines showing quantitative relative inhibition of colony formation as displayed in (**A**). * *p* < 0.05, ** *p* < 0.01, *** *p* < 0.001 and **** *p* < 0.0001.

To further validate the effects of tivantinib on NB proliferation, we performed colony formation assays using six NB cell lines. Our data show that tivantinib profoundly inhibited colony formation in all NB cell lines tested at a significantly lower dose (Figure 3). These data demonstrate that tivantinib has specific anti-proliferative effects against NB cells.

### 2.3. Tivantinib Triggers Apoptosis and Halts the Cell Cycle Progression in NB Cells

**Figure 4:**
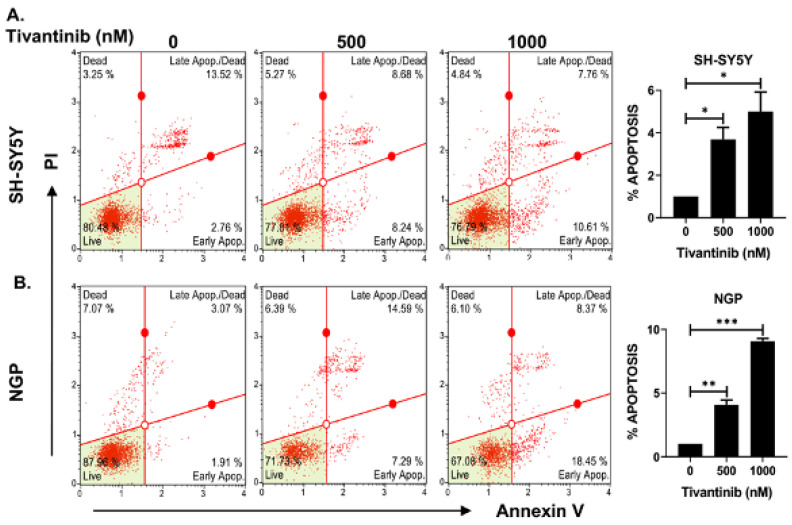
Tivantinib induces apoptosis in NB cells. Apoptosis assays in response to tivantinib treatment in NB cells. (**A**,**B**) Flow cytometer plots of apoptosis analysis and quantitative analysis of the percentage of early apoptosis cells in (**A**) SH-SY5Y and (**B**) NGP cells. *p < 0.05, ** *p* < 0.01, *** *p* < 0.001.

**Figure 5:**
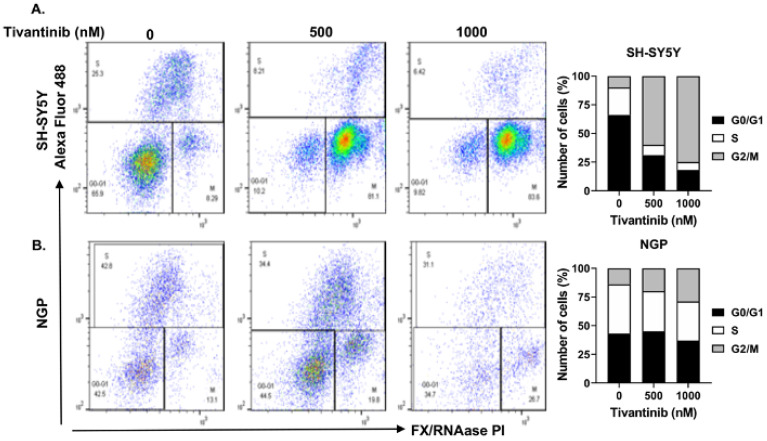
Tivantinib blocks NB cell cycle progression. Cell cycle assays in response to tivantinib treatment in NB cells. (**A**,**B**) Flow cytometer plots of cell cycle assays and quantitative analysis of the percentage of cells in each cell cycle phase of in (**A**) SH-SY5Y and (**B**) NGP cells.

To further investigate the mechanism through which tivantinib hinders the NB cell proliferation, we conducted functional assays of apoptosis and cell cycle phase determination using two distinct NB cell lines: NGP, a MYCN-amplified cell line, and SH-SY5Y, a MYCN non-amplified cell line. Our findings demonstrate that tivantinib robustly triggers early apoptosis in both the cell lines (~3.8-fold increase in SH-SY5Y and 9.7-fold increase in NGP) in a concentration-dependent manner, compared to control treatments involving placebo (DMSO) (Figure 4). Additionally, our analysis of cell cycle progression reveals that tivantinib effectively obstructs the advancement of NB cells into the G2/M phase in both cell lines, exhibiting dose-dependent behavior (Figure 5). Specifically, the cell cycle data indicate a substantial upsurge of cells in the G2/M phase by ~10-fold in SH-SY5Y and 2-fold in the NGP cell line. This is accompanied by a notable reduction in the proportion of cells in the S phase, which contrasts with the outcomes of the control treatments (Figure 5). These results emphasize the potency of tivantinib in restraining NB proliferation by impeding cell cycle progression at the G2/M phase and prompting apoptosis in NB cells, regardless of their MYCN status.

### 2.4. Tivantinib Inhibits NB Spheroid Tumor Growth

**Figure 6:**
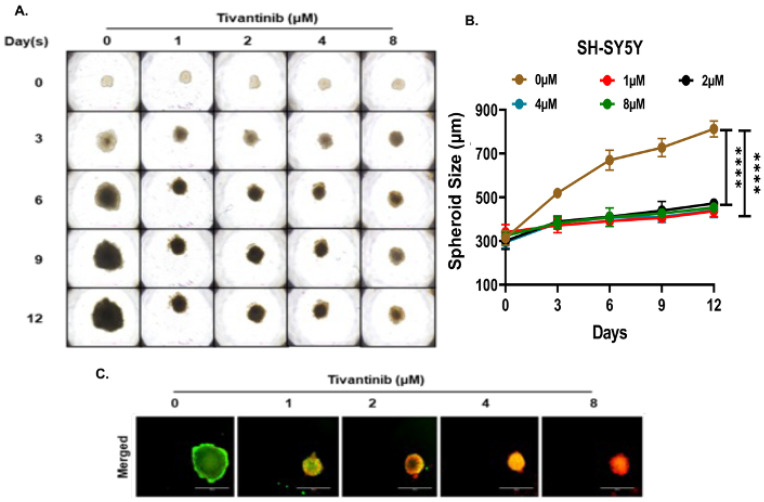
Tivantinib inhibits SH-SY5Y NB 3D spheroid tumor growth. (**A**) Representative images of NB 3D spheroidal tumors of MYCN non-amplified SH-SY5Y cells. (**B**) Quantitative 3D spheroid tumor growth in response to tivantinib treatment. (**C**) Representative fluorescent images stained with Calcein AM (Green; live cells) and EthD-III (Red; dead cells) fluorescence dyes. **** *p* < 0.0001.

**Figure 7:**
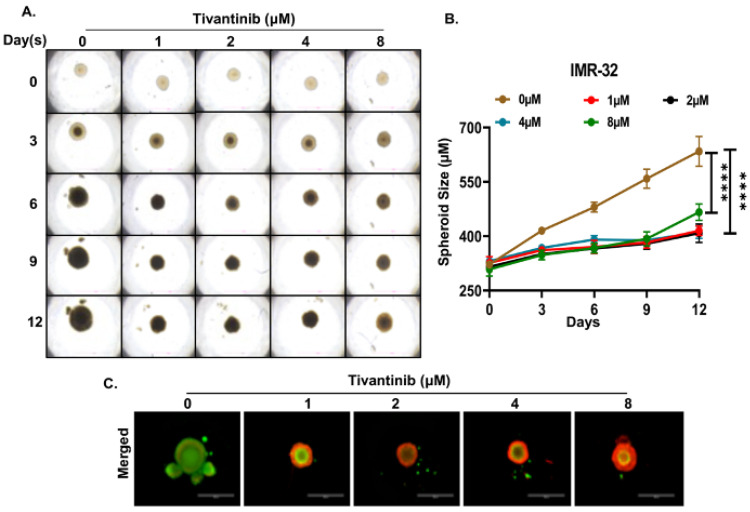
Tivantinib inhibits IMR-32 NB 3D spheroid tumor growth. (**A**) Representative images of NB 3D spheroidal tumors of MYCN amplified IMR-32 cells. (**B**) Quantitation of 3D spheroid tumor (**C**) Fluorescent images demonstrating live (Green) and dead cells (Red) in NB 3D spheroids. **** *p* < 0.0001.

To gain deeper insights into the effects of tivantinib in NB, we established NB 3D spheroid tumor models using two NB cell lines, SH-SY5Y and IMR-32, representing MYCN non-amplified and amplified status. These 3D spheroid tumors replicate the in vivo physiological growth patterns observed in solid tumor NB. We developed spheroids of comparable sizes and subjected them to various doses of tivantinib. Our findings reveal that even the lowest tivantinib concentration (1 µM) significantly inhibits the overall growth of NB 3D spheroid tumors in both SH-SY5Y and IMR-32 spheroid models (Figure 6A,B and Figure 7A,B). Furthermore, staining of the terminal day (day 12) spheroids with Calcein-AM (a marker for live cells) and EthD-III (a marker for dead cells) shows that tivantinib significantly and in a dose-dependent manner inhibits the cell viability of spheroids (Figure 6C and Figure 7C). These observations validate the efficacy of tivantinib in inhibiting NB 3D spheroid growth, which recapitulates the solid tumor growth pattern.

### 2.5. Tivantinib Suppresses the c-MET Signaling Cascade

**Figure 8:**
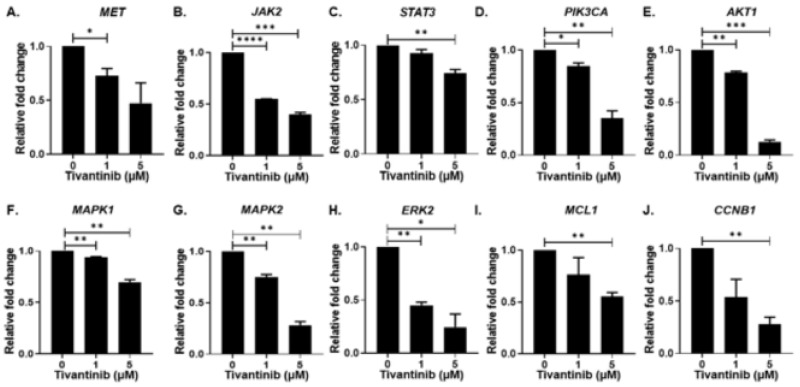
Tivantinib inhibits gene expression of MET and downstream regulators. Gene expression analysis of MET and downstream signaling pathway genes in response to tivantinib treatment. (**A**) *MET* (**B**) *JAK2* (**C**) *STAT3* (**D**) *PIK3CA* (**E**) *AKT1* (**F**) *MAPK1* (**G**) *MAPK2* (**H**) *ERK2* (**I**) *MCL-1* (**J**) *CCNB1*. * *p* < 0.05, ** *p* < 0.01, *** *p* < 0.001, **** *p* < 0.0001.

We further expanded our investigation to dissect the underlying molecular mechanism of tivantinib by evaluating c-MET signaling pathway genes and proteins in the NB SH-SY5Y cell line. Our gene expression analysis unveiled a concentration-dependent decrease in *MET* mRNA expression upon tivantinib treatment compared to the control treatment. Additionally, we found that tivantinib treatment exerts inhibitory effects on various genes within the JAK/STAT pathway, specifically *JAK2* and *STAT3*, as well as genes in the PI3K/AKT pathway, including *PIK3CA*, *AKT1*, as well as Ras/Raf pathway including *MAPK1*, *MAPK2*, and *ERK2*. Notably, tivantinib also inhibited the expression of an anti-apoptotic gene, MCL1, and an essential cell cycle regulator gene, *CCNB1* (Cyclin-B1), in a concentration-dependent manner (Figure 8).

**Figure 9:**
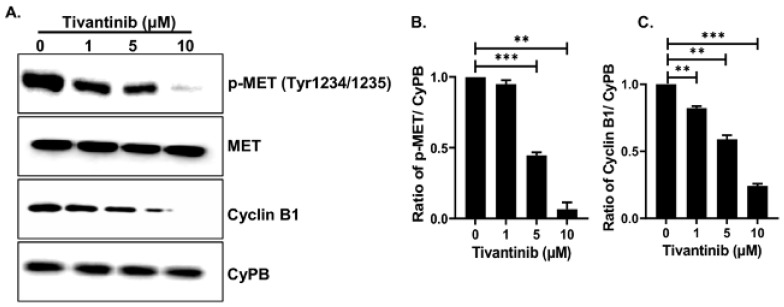
Tivantinib inhibits MET and Cyclin B1 proteins. (**A**) Western blot analysis of total MET, pMET (Tyr1234/1235), and Cyclin B1 in response to tivantinib treatment. (**B**,**C**) Densitometric analysis of protein bands as shown in (**A**). (**B**) pMET and (**C**) Cyclin B1. ** *p* < 0.01, *** *p* < 0.001.

Further, our immunoblotting analysis provided additional notable evidence that tivantinib effectively blocks c-MET phosphorylation at the active Tyr1234 site without altering the total MET levels, as compared to the control treatment and the loading control (Figure 9A,B). Additionally, we found that tivantinib inhibits the cell cycle regulator protein Cyclin B1 in a concentration-dependent manner (Figure 9A,C). These immunoblots were quantified using densitometric analysis, which showed a 10-fold inhibition of c-MET Tyr1234 phosphorylated and about 5-fold inhibition of Cyclin B1 protein levels with ten µM tivantinib treatments, respectively, in comparison to loading control CyPB and control treatments (Figure 9B,C). These findings revealed the impact of tivantinib in hampering the c-MET pathway and cell cycle regulator at both mRNA and protein levels.

## 3. Materials and Methods

### 3.1. Cell Culture and Reagents

Human NB cell lines, including MYCN amplified (NGP, LAN-5, IMR-32) and MYCN non-amplified (SH-SY5Y, SK-N-AS, CHLA-255), along with control fibroblast cell lines (WI-38, NIH-3T3, COS-7), were cultured and maintained as described previously [27]. NB and fibroblast cell lines were authenticated via short-tandem repeat analysis for genotyping and examined for mycoplasma within the last two months. Primary antibodies cyclin-B1 (626901) and Met (689902) were purchased from BioLegend (San Diego, CA, USA). Primary antibodies p-Met (3077) and anti-cyclophilin B (43603), and HRP-conjugated anti-rabbit IgG secondary antibody (7074) were purchased from Cell Signaling Technology (Danvers, MA, USA). Tivantinib was purchased from MedChem Express (Monmouth Junction, NJ, USA).

### 3.2. Dataset Analyses

Publicly accessible R2: Genomic Analysis and Visualization Platform was used to analyze 1235 primary NB patient samples from various datasets of SEQC (n = 498), Kocak (n = 649), and Versteeg (n = 88). R2: Genomic Analysis platform provides multi-parametric analysis of individual primary tumors and gene expression profiles. We also analyzed the Cancer Cell Line Encyclopedia database of Broad Institute and utilized the DepMap Portal tools to analyze the correlation of NB cell lines with MET expression levels.

### 3.3. Cell Proliferation and Clonogenic Assays

Cell proliferation assays were performed using 3-(4,5-Dimethylthiazol-2-yl)-2,5-Diphenyltetrazolium Bromide) dye (L11939; Alfa Aesar, Haverhill, MA, USA), as described previously after exposing different NB cells and control fibroblast cells to increasing concentrations of tivantinib for 72 h [27]. Data were analyzed, and IC_50_ values were calculated using GraphPad Prism (Prism 9, San Diego, CA, USA). Clonogenic assays were performed using crystal violet solution (0.2%) as described previously [27]. Colony counting software (OpenCFU ver. 3.8) was used to count and visualize the stained colonies.

### 3.4. Cell Cycle and Apoptosis Assays

Cell cycle analysis was performed using the FxCycle™ PI/RNase Staining Solution (F10797; ThermoFisher Scientific, Waltham, MA, USA) and Click-iT™ Plus EdU Alexa Fluor™ 488 Flow Cytometry Assay Kit (C10633; ThermoFisher Scientific,). As described previously, Muse Annexin V and Dead Cell Kit (MCH100105; Luminex Corp, Austin, TX, USA) were used to conduct apoptosis experiments [27]. Both cell cycle and apoptosis experiments were conducted after treating NB cells with escalating doses of tivantinib for 12 h by using Attune Nxt flow cytometer (ThermoFisher Scientific) and Muse flow cytometer (Luminex), respectively. Flow cytometry data were analyzed using Flow Jo (BD Biosciences, Ashland, OR, USA) and Muse (Luminex) software [27].

### 3.5. RNA Extraction and Quantitative Real-Time RT-PCR

RT-qPCR technique was used to analyze gene expression after NB cells were exposed to escalating doses of tivantinib for 12 h. Total RNA was extracted using RNeasy plus mini kit (74134; Qiagen, Germantown, MD, USA), followed by cDNA synthesis using a high-capacity cDNA reverse transcription kit (4368814; ThermoFisher Scientific) as described previously and according to the manufacturer instructions [27]. Further, RT-qPCR reactions for specific genes were carried out using the QuantStudio 3 Real-Time PCR System (ThermoFisher Scientific) using the SYBR Green dye method (4385610; ThermoFisher Scientific). GAPDH was used as a housekeeping gene to normalize the expression of each gene. Gene-specific RT-qPCR primers were used in this study and are listed in Table 1.

### 3.6. Immunoblotting Assays

Immunoblotting assays were conducted as described previously [27]. Briefly, SH-SY5Y cells were subjected to escalating doses of tivantinib for 24 h, followed by extraction and quantification of protein. The protein samples were separated on a 4–20% gradient SDS-PAGE gel and transferred onto the PVDF membrane, followed by blocking and probing with respective primary and HRP-conjugated anti-rabbit or anti-mouse secondary antibodies, as reported earlier [27]. Protein samples were normalized using cyclophilin B (CyPB) as a loading control.

### 3.7. Spheroid Formation Assay

As reported previously, the 3D spheroid 96-well microplates (4515; Corning, Corning, NY, USA) were used to conduct the 3D spheroid tumor experiments [27]. NB spheroids were developed, randomized, and treated with various concentrations of tivantinib. Spheroid images were captured, and tumor size and the number of live and dead cells were analyzed, as reported in our previous studies [27].

### 3.8. Statistical Analysis

A two-tailed student *t*-test was conducted to investigate the statistical significance of drug treatment groups. The significance of the fold change in each gene’s expression was evaluated using the student’s *t*-test. In this study, each experiment was performed three times with three technical replicates.

## 4. Discussion

N-methyl-N′-nitroso-guanidine human osteosarcoma transforming gene, often referred to as *MET*, is a tyrosine kinase. MET is instrumental in regulating various cellular activities such as cell proliferation, migration, organogenesis, embryogenesis, angiogenesis, and tissue regeneration [28,29,30]. The hyperactivity of c-MET and HGF contributes to tumor progression, metastasis, and invasion in various cancers and correlates with advanced cancer stages, poor overall patient survival rate, and prognosis [6,28,31]. We also observed similar results by analyzing primary NB patient samples from three different datasets and found that the *MET* expression correlates with poor NB patient survival, advanced stages, disease relapse, and worst outcomes.

Inhibitors of c-MET showed potent anti-tumor activity against various cancers, including gastric cancer and hepatocellular carcinoma (HCC) [30,32,33]. Tivantinib and capmatinib are small-molecule c-MET inhibitors designed to target c-MET receptors and are currently in the early phase of clinical studies in treating a plethora of cancers, including metastatic NSCLC, non-squamous non-small cell neoplasm of the lung, metastatic colorectal cancer, advanced HCC, and stage IV prostate cancer [34,35]. Recently, c-MET inhibitors Tepotinib and Capmatinib received FDA approval for adult metastatic NSCLC patients with MET exon 14 skipping alterations [35,36]. Tivantinib, either alone or in combination, is currently under various phases of clinical investigation (phase I/II and phase III) to treat divergent tumors, including advanced solid tumors, NSCLC, gastroesophageal cancer, recurrent malignant mesothelioma, pancreatic neoplasms, renal cell cancer (NCT01178411, NCT01069757, NCT01611857, NCT01861301, NCT00558207, NCT01688973). However, tivantinib showed no antitumor effect in the phase III MET-high advanced hepatocellular carcinoma (METIV-HCC trial) [37]. Our findings are consistent with those that revealed MET gene suppression, which is one of the important attributes in NB. However, tivantinib’s effects on NB have yet to be clinically explored. Still, more in-depth preclinical models and clinical trials should be conducted to assess the potential of tivantinib as a drug to suppress MET in NB patients.

Obstruction of the MET/HGF pathway has been delineated to inhibit NB [38]. In our study, tivantinib showed a specific anti-tumor effect in NB, especially given its selective effect on all the NB cell lines tested irrespective of the MYCN amplification status and without impacting non-cancerous fibroblast cells. Tivantinib improved the survival of HCC patients by 2.7 months [39]. In contrast, Kobayashi et al., demonstrated that HCC patients have less sensitivity toward tivantinib [40]. Contrasting reports on tivantinib’s role in HCC underline the need for cautious interpretation and comprehensive clinical trials. Wu et al. reported that tivantinib’s effectiveness might be compromised due to aberrant activation of the ABCG2 receptor, which is associated with multi-drug resistance in numerous cancers [41]. Tivantinib is reported as a more prominent GSK3α and GSK3β inhibitor and a weak MET inhibitor in acute myeloid leukemia [10]. Tivantinib has been reported to inhibit cancer cell proliferation either through c-MET inhibition, microtubule inhibition, or both pathways [22,23,24,26]. The ability of tivantinib to induce G2/M phase arrest by disrupting microtubule polymerization offers a strategic avenue to halt tumor growth [23]. Our findings further substantiate this effect, as evidenced by the significant accumulation of NB cells in the G2/M phase upon tivantinib treatment. The consistent downregulation of Cyclin B1, a crucial regulator of the G2/M transition in the cell cycle, further delineates tivantinib’s anti-proliferative mechanism. This dose-dependent inhibition mirrors results observed in HCC, demonstrating that tivantinib’s effects on cell cycle regulation might be a universal mechanism for various cancer types [42]. Similarly, in breast cancer, regulators such as TRPM7 and anesthetic treatment have been shown to regulate cell viability, cell cycle, and cell migration [43,44]

ALK mutations have been observed in 5–8% of NB patients in one of the three tyrosine kinase domains, including R1275, F1174, and F1245. In this study, SH-SY5Y is an ALK (F1174L) mutated MYCN-non-amplified cell line, and LAN-5 is an ALK (R1275Q) mutated MYCN-amplified cell line, while other cell lines are ALK wild-type [27]. Crizotinib, a first-generation non-selective tyrosine kinase inhibitor, has been reported to inhibit both ALK and MET receptors [45]. MET inhibitors have been reported to be an efficient treatment regimen combined with other drugs in various cancers, including colorectal, gastric, and pancreatic [46]. Some studies have reported that combining MET inhibitors with EGFR tyrosine kinases may overcome drug-resistance problems [47]. A phase III clinical trial was conducted in advanced NSCLC patients by comparing erlotinib and tivantinib versus erlotinib and placebo [48,49]. Combining MET and PI3K inhibition is efficacious in malignant pleural mesothelioma [50]. Pazopanib and tivantinib modulated the VEGF and MET levels in refractory solid tumors [51]. Alterations in MAPK pathway genes with MET mutations decreased the MET inhibitor sensitivity in lung cancer [52]. A combination of tivantinib and zoledronic acid prevented the tumor bone engraftment and bone metastasis in breast xenograft models [53].

Our data also reveal that tivantinib inhibits the expression of the anti-apoptotic gene MCL-1 and induces apoptosis in NB cells. Similar observations were reported for inducing apoptosis in drug-resistant NSCLC cell lines by inhibiting the anti-apoptotic genes [54]. Additionally, Tivantinib’s mechanism of action is known to obstruct several cell signaling pathways, including AKT, Ras/Raf, STAT, GSK3α/β, JNk/c-jun, and the NF-κB pathway [26,55,56,57,58]. Similarly, our gene expression and immunoblotting analysis showed tivantinib-mediated concentration-dependent inhibition of c-MET phosphorylation at the active site Tyr1234 that further resulted in the inhibition of cell signaling pathway genes *JAK2*, *STAT3*, *PI3KC2A*, *AKT1*, *MAPK1*, *MAPK2*, and *ERK2*. We observed MET expression at both mRNA and protein levels in the NB SH-SY5Y cell line, in contrast to the previous report of no c-MET expression [6]. Similarly, high levels of MET expression were observed in gastric and NSCLC cancer models that drive tumor growth and metastasis [31,44]. Tivantinib, in combination with other tyrosine kinase inhibitors such as erlotinib and sorafenib, showed a promising therapeutic approach for EGFR-Mutant NSCLC and HCC, respectively [59,60]. Tivantinib had intriguing anti-tumor growth activity and pro-apoptotic and anti-proliferative properties in HCC, multiple myeloma, breast, and pancreatic in vivo tumor models [24,61,62]. Spheroid tumors that grow as in vitro 3D tumors mimic in vivo solid tumor development patterns and growth and are used for in vitro drug screening and testing [63]. We demonstrated that tivantinib inhibits NB 3D spheroid tumor formation and progression by suppressing the tumor-living cells in a concentration-dependent manner.

Our study concludes by highlighting the effects of tivantinib in reducing NB growth by specifically inhibiting the phosphorylation and activation of c-MET. Tivantinib specifically inhibited the [43,44] of NB cells in both 2D cell culture and 3D spheroid tumor models. To further trigger apoptosis and halt cell cycle progression in NB, tivantinib inhibits the expression of many oncogenic cell signaling pathways and Cyclin B1, a cell cycle regulator. Overall, our pre-clinical data suggest that c-MET inhibitor tivantinib could be an effective therapeutic strategy for treating NB. However, additional studies are required to validate its clinical efficacy and assess its potential as part of a comprehensive therapeutic strategy for high-risk NB. Our findings also emphasize the importance of further developing c-MET targeted therapy approaches and integrating them with current therapies for efficiently managing high-risk NB.

## Figures and Tables

**Table 1:** RT-qPCR primers used in the study.

Gene	Forward Primer (5′-3′)	Reverse Primer (5′-3′)
*MET*	AGTGGGAATTCTAGACACATTTCA	CATTCAAGAATACTGTTTGACACACTT
*STAT3*	CCCTCAGCAGGAGGGCAGTT	TCACATGGGGGAGGTAGCACA
*JAK2*	GCAGGCAACAGGAACAAGAT	CCATTCCCATGCAGAGTCTT
*PI3KCA*	CGCATTTCCACAGCTACACC	AGCCATTCATTCCACCTGGG
*AKT1*	GCACAAACGAGGGGAGTACA	AAGGTGCGTTCGATGACAGT
*MCL1*	TCGTAAGGACAAAACGGGAC	CATTCCTGATGCCACCTTCT
*ERK2*	ACGGCATGGTTTGCTCTGCTTATG	TCATTTGCTCAATGGTTGGTGCCC
*MAPK1*	TACACCAACCTCTCGTACATCG	CATGTCTGAAGCGCAGTAAGATT
*MAPK2*	GGCAGCTACCTCAGGAATGAC	CCAGTGGCATGGTAAATCTCC
*CCBN1*	AAGAGCTTTAAACTTTGGTCTGGG	CTTTGTAAGTCCTTGATTTACCATG
*GAPDH*	CACCATCTTCCAGGAGCGAG	TGATGACCCTTTTGGCTCCC

## Data Availability

R2: Genomic Analysis and Visualization Platform (https://hgserver1.amc.nl/cgi-bin/r2/main.cgi accessed on 10 March 2024). Cancer Cell Line Encyclopedia database of Broad Institute (https://sites.broadinstitute.org/ccle/tools accessed on 15 July 2024). DepMap Portal tools (https://depmap.org/portal/ accessed on 15 July 2024).
